# Polymer Nanoparticles Applied in the CMP (Chemical Mechanical Polishing) Process of Chip Wafers for Defect Improvement and Polishing Removal Rate Response

**DOI:** 10.3390/polym15153198

**Published:** 2023-07-27

**Authors:** Wei-Lan Chiu, Ching-I Huang

**Affiliations:** Institute of Polymer Science and Engineering, National Taiwan University, Taipei 10617, Taiwan; d99549014@ntu.edu.tw

**Keywords:** chemical mechanical polishing, polymer nanoparticle, particle shape, particle size, particle solid content, colloidal silica, fume silica, polishing removal rates, polishing defect counts, polishing uniformity, aggregation ratio, block copolymer, polystyrene

## Abstract

Chemical mechanical planarization (CMP) is a wafer-surface-polishing planarization technique based on a wet procedure that combines chemical and mechanical forces to fully flatten materials for semiconductors to be mounted on the wafer surface. The achievement of devices of a small nano-size with few defects and good wafer yields is essential in enabling IC chip manufacturers to enhance their profits and become more competitive. The CMP process is applied to produce many IC generations of nanometer node, or those of even narrower line widths, for a better performance and manufacturing feasibility. Slurry is a necessary supply for CMP. The most critical component in slurry is an abrasive particle which affects the removal rates, uniformity, defects, and removal selectivity for the materials on the wafer surface. The polishing abrasive is the source of mechanical force. Conventional CMP abrasives consist of colloidal silica, fume silica or other inorganic polishing particles in the slurries. We were the first to systematically study nanoparticles of the polymer type applied in CMP, and to compare traditional inorganic and polymer nanoparticles in terms of polishing performance. In particular, the polymer nanoparticle size, shape, solid content dosing ratio, and molecular types were examined. The polishing performance was measured for the polishing removal rates, total defect counts, and uniformity. We found that the polymer nanoparticles significantly improved the total defect counts and uniformity, although the removal rates were lower than the rates obtained using inorganic nanoparticles. However, the lower removal rates of the polymer nanoparticles are acceptable due to the thinner film materials used for smaller IC device nodes, which may be below 10 nm. We also found that the physical properties of polymer nanoparticles, in terms of their size, shape, and different types of copolymer molecules, cause differences in the polishing performance. Meanwhile, we used statistical analysis software to analyze the data on the polishing removal rates and defect counts. This method helps to determine the most suitable polymer nanoparticle for use as a slurry abrasive, and improves the reliability trends for defect counts.

## 1. Introduction

Chemical mechanical polishing (CMP) is a method of wafer surface planarization for the integrated circuit (IC) chip manufacturing process. CMP processes involve the removal and flattening of wafer surface materials, including metals, dielectrics, polymers, and other thin films associated with semiconductor manufacturing. The acquisition of fully flat wafer surfaces obtained through planarization is important for IC production technologies [1,2,3,4,5], which aim to produce IC device of increasingly smaller sizes on wafers. These devices’ sizes are defined according to the node generation of nanometers, such as N10, N5, and N3 [6,7,8].

In this article, we introduce the mechanism of CMP processes and the significant factors impacting the abrasives used in CMP slurry. In particular, we applied polymer particles to a CMP slurry, being the first study to systematically analyze and compare the polishing defect results of such materials with those of inorganic particle slurries. Based on the defect counting values and polishing removal rates, we propose suitable polymer abrasives that can improve the yields of IC chips in wafer fabrication and manufacturing.

**Figure 1:**
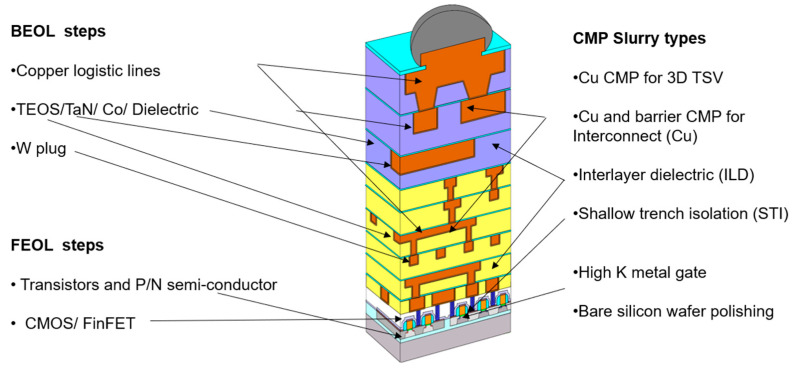
A schematic plot of semiconductor fabrication for wafers that can be classified as including front-end-of-line (FEOL) production steps, in white, and back-end-of-line (BEOL) steps, indicated by the purple and yellow regions [9].

Figure 1 shows a schematic plot of semiconductor fabrication for wafers that can be classified into a front-end-of-line (FEOL) step, indicated by the white regions with interior brown segments, and back-end-of-line (BEOL) steps, indicated by the purple and yellow regions with interior brown segments [9]. These are the production phase steps. The FEOL step involves the fabrication of the devices on silicon wafers using non-metal materials or semiconductors, and the BEOL step mainly involves fabrication with metal materials that form interconnectors or circuits on the wafers for transistor devices [10]. The metals applied for IC manufacturing are usually tantalum (Ta), tantalum nitride (TaN), cobalt (Co), and copper (Cu). The metal stack sequence is based on the scheme design [11,12,13]. The metal materials are applied to fill in grooves or holes in the dielectric layers, such as TEOS and low-K materials (BD2). In order to connect the metal wires and metal connectors by tungsten plug [14] between the covered conductive layers and reduce electron migration diffusion, the barrier metals are generally formed before the Cu lines are deposited. Currently, Co, Ta, and TaN are deemed to be suitable barrier metal species because of their good adhesion with dielectric materials and superior electro-resistance [15,16]. As a result of the barrier metal’s good capacity for adhesion to Cu seed crystals, it also ensures the filling of the grooves of the BEOL phase with the deposited copper [17].

**Figure 2:**
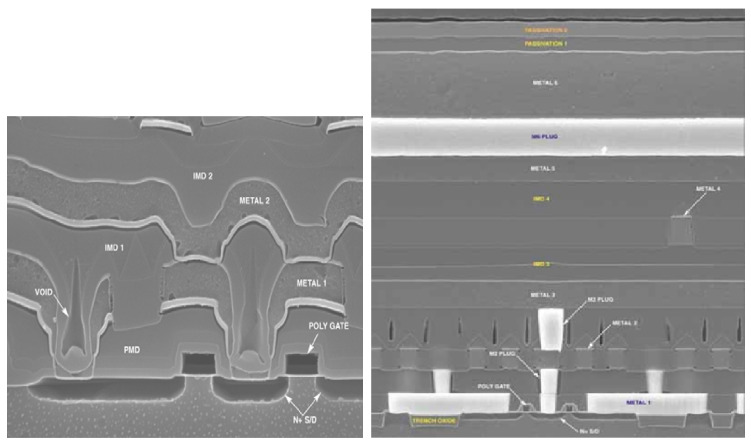
A cross-sectional image of integrated circuits and transistor components on the wafer surface, with and without chemical mechanical polishing [20].

**Figure 3:**
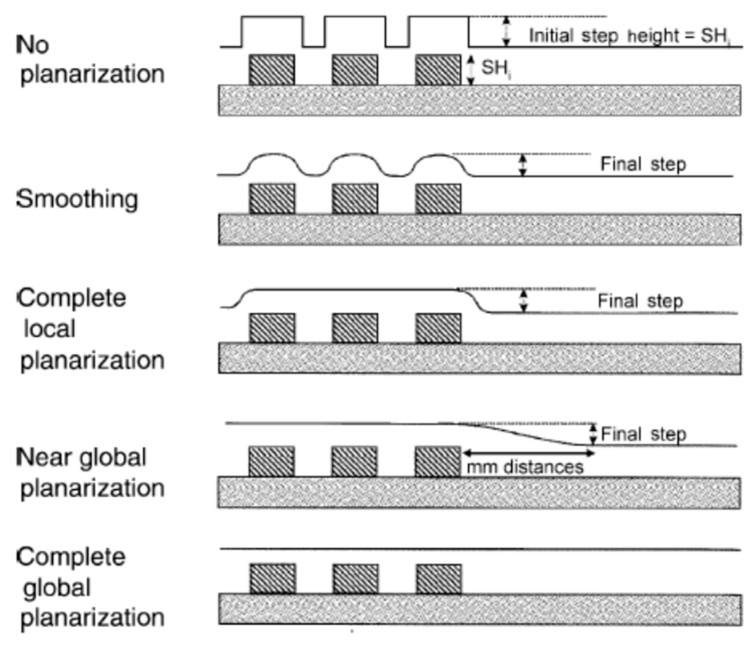
Illustration of the CMP process indicating the degrees of planarization for the deposited material [26].

In order to achieve integration for IC fabrication, as shown in Figure 1, the CMP process is used to planarize metal that is unevenly deposited on the wafer surface, allowing one to easily create orderly stacks. CMP uses a combination of chemical and mechanical forces to achieve planarization [18,19], and the difference for planarization in CMP is shown in Figure 2, in which the cross-section images depict the IC for the transistor devices and conductive copper lines on the wafer surfaces. The figure shows the results with and without chemical mechanical polishing [20]. Due to the need for good-quality planarization in CMP, one must design a high-performance liquid solution called a polishing slurry. The formulation of CMP slurry is a complex science in which the chemical force of the chemical ingredients been dosed in water by tuning suitable pH [21] and the mechanical force of the synergy grinding particles are influential [22]. To obtain proper material removal rates (MRR), good uniformity and low defect counts are important for slurries [23,24,25]. The material removal rate is defined as the thickness removal amount/per minute (Å/min) for CMP, considered as the target value. The phenomenon of polished wafer surface planarization is defined as non-uniform (N.U.). As illustrated in Figure 3, the MRR target value is used to evaluate the step height reduction for uneven materials that are deposited on the IC devices of wafers. The uneven initial step height (SH) is gradually reduced during the polishing processes. In the final stage, the aim is to complete the global planarization process [26].

**Figure 4:**
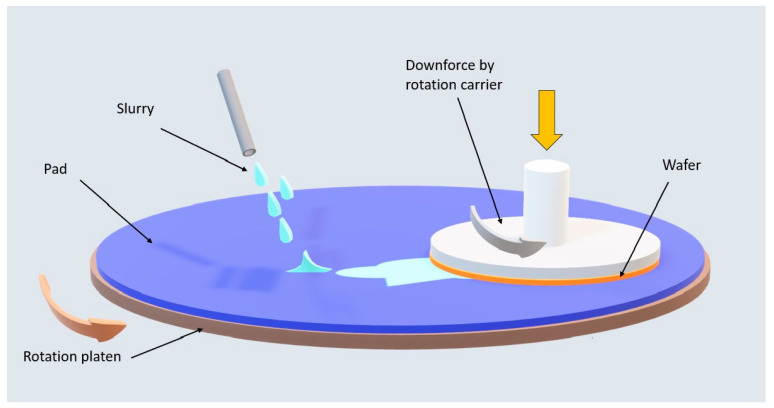
Schematic diagram of contact between the wafer, the rotation polishing pad on the platen, and the slurry during the CMP process.

**Figure 5:**
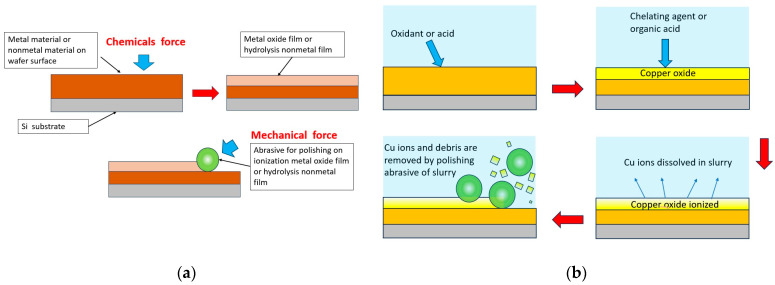
(**a**) The oxide metal film is removed with the mechanical abrasive force caused by the sliding of the abrasive. (**b**) The copper oxide is ionized by chemicals with abrasives that provide simultaneous polishing through mechanical force.

Figure 4 demonstrates the polishing scheme for a wafer assembly based on the polishing head and slurry dosing. The wafer is polished by simultaneously applying slurry between the assembled wafer in the carrier head and the pad on the rotation platen. The polishing equipment provides the downforce (P) for the polishing head and the rotation speed (V) for the polishing platen and polishing head. Preston et al. [27] developed an early model of the material polishing mechanism of wafer surface materials. Our series of tests in this article were based on the same polishing recipe setup. Figure 5 illustrates the copper polishing processes used to explore the synergetic behavior of the chemical and mechanical forces. Kaufman et al. [28] proposed a theoretical mechanism of the removal rate based on the pressure and relative velocity applied between the wafer and pad. As shown in Figure 5a, the chemical reaction layer formed in the chemical reaction between the wafer and the oxide metal film is removed, together with the mechanical abrasive force caused by the sliding of the abrasive [29]. The general removal mechanism is such that after the oxide layer is formed, it is removed as a result of physical friction generated by the mechanical force. As shown in Figure 5b, taking copper metal as an example, the copper is oxidized with acid-containing oxidants, and the passivation layer is a chemical reaction layer that produces a stable copper oxide on the surface to be polished. Then, the copper oxide is ionized by chemicals with abrasives that provide simultaneous polishing through mechanical force. Paul et al. [30] improved the mechanistic model using simple kinetic energy expressions to quantitatively describe the chemical reactions of the material removal rates. This model is based on the notion that the complex chemical reactions and mechanical grinding processes are separable.

In the traditional methods for polishing abrasives, such as the use of fume silica or aluminum oxide for CMP, polishing defects are easily generated. There are many types of defects are induced during CMP processes. The common defects include scratches, pinholes, pits, residues, and other undefined defects in various detected positions. Defects on the surfaces of the wafers have a decisive impact on the commercial production of IC wafers. As a defect affects the yield of every chip on the wafer, the yield affects the competitiveness of the industry and the companies within it. In order to achieve reasonable removal rates and low defect counts performance, we must understand the principles of CMP processes. CMP results from complex interactions between chemical and mechanical forces [31]. Since polishing defects are the most crucial aspect of CMP, we applied polymer nanoparticles to investigate CMP removal rates and defect performance in this study. To our knowledge, we were the first to conduct systematic research in applying polymer nanoparticles to metal-stage (BEOL) CMP slurries for defect improvement.

**Figure 6:**
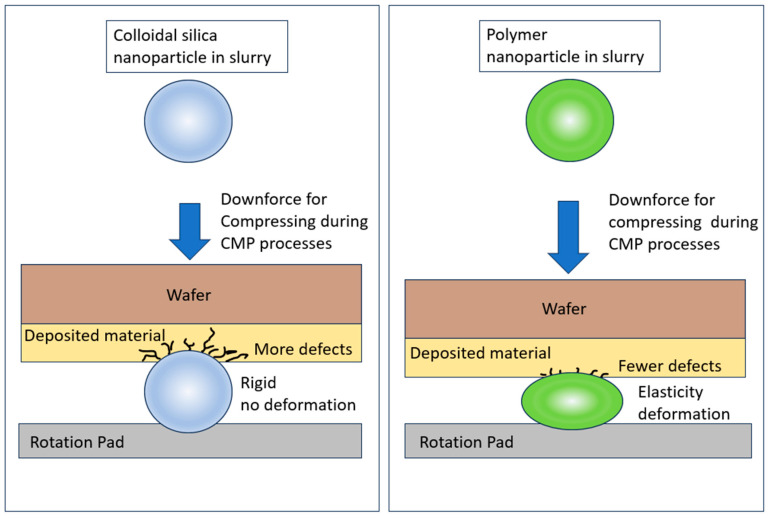
Polymer nanoparticles, as abrasives, were compressed during CMP processes to reduce the numbers of defects and scratches, and compared with the performance of silica.

Materials deposited on wafers have different physical hardness properties and chemical resistance abilities, leading to differing polishing performance. Compared with inorganic hard nanoparticles, polymer nanoparticles provide a relatively gentle mechanical force mechanism, and viscoelastic particles reduce the occurrence of polishing defects. As shown in Figure 6, polymer nano-abrasive particles are compressed during CMP processes to reduce defects and scratch counts, as compared with the results achieved using traditional slurry abrasives formed from inorganic particles such as silica. Since silica is a rigid inorganic particle, it produces great friction force during CMP processes. The mechanical friction force also acts in synergy with the effect of the chemical force to generate specific polishing removal rates. From the microscopic point of view, softer particles have better uniformity and lower total counts of polishing defects such as scratches.

In this study, the CMP processes were investigated in relation to the mass transfer phenomenon, with the interface between the polymer nanoparticles and wafers affecting chemical and mechanical forces deriving from the intrinsic physical properties of the polymer nanoparticles. Therefore, we proposed a series of tests aiming to examine factors related to polymer nanoparticle polishing removal rates and total defect counts. First, we carried out a comparison between the polishing performance of traditional silica and polymer nanoparticles. Second, we used a series of polystyrene nanoparticles with different solid contents of nanoparticles, particle shapes, and particle sizes. These polymer nanoparticles were obtained using the emulsifier-free method. Since there is no presence or residue of an emulsifier, the results of the CMP polishing data and our discussion excluded the interface effect from which the surfactant or emulsifier is derived. We used these polymer nanoparticles to formulate different slurries for polishing tests, and then analyzed the polishing data. We also used various types of copolymer molecules of nanoparticles to compare their polishing removal rates and total defect count performance. Meanwhile, these polishing performance data were utilized in a software-based statistical analysis to identify suitable polymer nanoparticles for CMP applications.

## 2. Materials and Methods

The polymer nanoparticles were formed using polystyrene molecules, PMMA, and 4 kinds of copolymers with a number-averaged molecular weight (Mn), weight-averaged molecular weight (Mw), and PDI of around 2 × 10^4^~2.5 × 10^4^, 7 × 10^4^~8.75 × 10^4^, and 3.5, respectively, using GPC equipment. The particles were synthesized with the emulsifier-free polymerization method. Then, we employed DLS (Dynamic Laser Scattering: Marlven), SEM, and BET to obtain the particle size distributions, shapes, and sizes, respectively. The DLS particle size results were all single-peak distributions, and the particle distribution index results indicated a high quality, according to the software analysis. The primary particle size (D1) was calculated for the surface area based on nonporous particles, with a single-point BET (Brunauer–Emmett–Teller) method, D1 [nm] = 2727/(SSA [m^2^/g]). The secondary particle size (D2) is the mean particle size [nm], calculated based on the scattering intensity of DLS (Dynamic Laser Scattering). The aggregated ratio (D2/D1) is the expression of the particle shape.

Table 1 lists the size distributions and shapes of all the nanoparticles. We formulated these nanoparticles into slurries to conduct CMP polishing tests. In this research, we focused on the polymer nanoparticle intrinsic polishing performance. Therefore, the essential components were simplified, and excluded some chemical dosing that are applied in slurry formulations. The chelating agent was citric acid, at 0.8%, used to chelate the oxidized copper. The chelated copper oxide could be ionized, and then polished. The testing of the slurries’ pH was conducted at 10.5 through KOH solution tuning. The oxidizer was H_2_O_2_ at a 1% absolute dosing ratio. No other metal inhibitors or surfactants were dosed in the slurries. In the series of CMP tests and in all figure captions, the particle size descriptions are all based on the size of D2.

**Figure 7:**
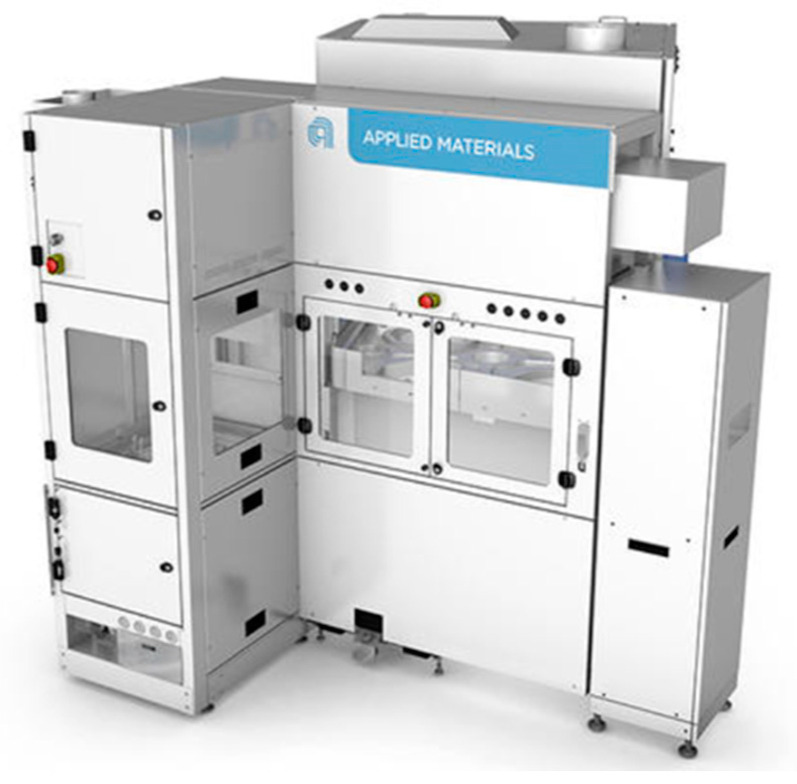
Chemical mechanical polishing (CMP) tool equipment.

Figure 7 displays the CMP polishing tool equipment, called Mirra Mesa, manufactured by the company Applied Materials. It is commercialized CMP equipment for 8-inch and 12-inch wafers. As can be seen here, the equipment comprises a single polishing platform and a single polishing head, with two-way or one-way wafer entry.

In the first step before the series of polishing tests, we set up the polishing parameters shown in Table 2 according to the standard polishing procedure for the Mira Mesa polishing equipment. We called this setup a polishing recipe. With the same polishing recipe, the polishing results are comparable. The reader can refer to the Introduction for the polishing pressure (P) and carrier head downforce pressure for wafers and the polishing speed (V) with respect to the platen rotation speed/wafer carrier head rotation speed. The supplied quantity of slurry is called the slurry flow rate (mL/min). After the polishing processes, all of the polished wafers were subjected to a post-cleaning procedure, in order to clean the slurry and residues on the polished wafer surfaces. We set up the standard post-cleaning tool and the cleaning solution based on deionized high-purity water as a benchmark for all of the polishing tests. Table 3 shows 8-inch wafers for CMP polishing. The four kinds of substrates were deposited on the wafer surfaces accordingly.

**Figure 8:**
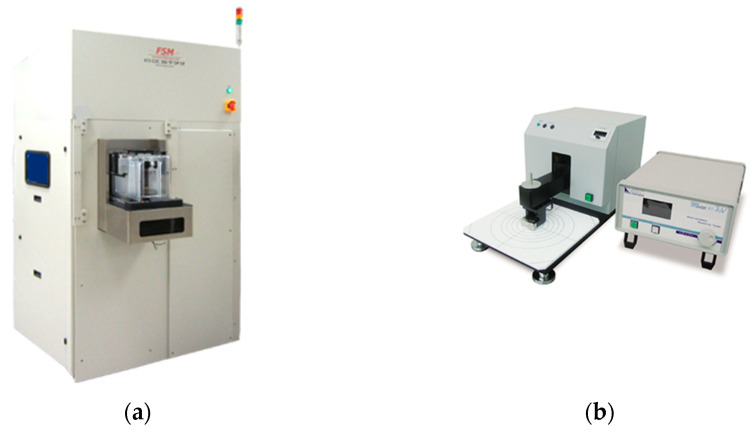
Wafer thickness measurements of non-metallic and metallic surfaces via (**a**) spectroscopic ellipsometry and (**b**) a 4-point probe, respectively.

We used spectroscopic ellipsometry to measure the thickness of a non-metallic material blanket wafer, as shown in Figure 8a. Based on the optical principle, the elliptical instrument is used to measure the thin film thickness of non-metallic materials. The equipment was applied to measure the thickness before CMP polishing and after CMP polishing to obtain the removal rates. Figure 8b shows the four-point probe instrument used to measure the thickness of the metal materials. This instrument applies the metal conductivity and the eddy current of the four-detection-point probe to measure the electrical resistance, which is correlated with the depth of the metal on the surface of the wafer. It can automatically calculate the thickness of the metal on the wafer.

**Figure 9:**
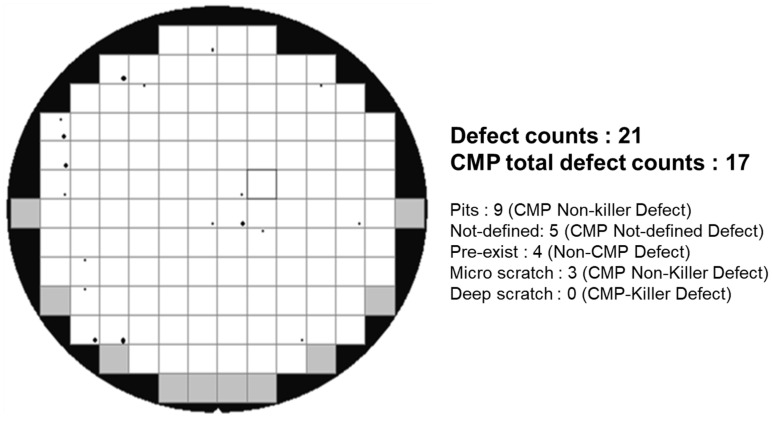
Example of results obtained through KLA automatic defect detection, indicating the defect types and the total number of defects.

A KLA defect analyzer was applied for the optical scanning of the entire wafer surface, and to record various defect images with the analysis software. The analyzer can automatically perform image identification using its internal image database library to obtain the types of defects and the total numbers of defects, as shown in Figure 9. The KLA tool can classify the types of defects, such as pits (non-killer defects); Not-defined(Not-defined defects); pre-exist defects(non-CMP defects); micro-scratches (non-killer defects); and deep scratches (CMP-killer defects).

## 3. Results and Discussion

### 3.1. Comparison by Nanoparticle Solid Content of Traditional Silica Nanoparticles and Polymer Nanoparticles for CMP Polishing Performance

**Figure 10:**
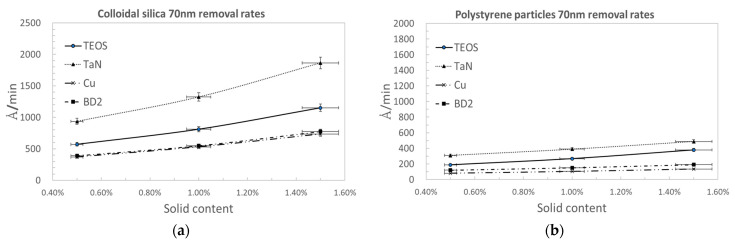
Removal rates as a function of solid contents of 0.5%, 1%, and 1.5%, according to a nanoparticle size of 70 nm for (**a**) traditional colloidal silica and (**b**) polystyrene particles.

We selected polymer nanoparticles of the same particle size for a comparison with traditional silica nanoparticles used for CMP polishing tests. The shape of the nanoparticles was round, being bead-like. The comparison was based on nanoparticles of the same size of 70 nm and the same shape. The polishing removal rates are demonstrated for four kinds of wafer substrates of TEOS, TaN, Cu, and BD2 (dielectric materials). Figure 10a,b display the polishing removal rates as a function of the solid content of colloidal silica and PS nanoparticles, respectively. As we can clearly see, the polymer nanoparticles show lower polishing removal rates for all the material substrates than the inorganic nanoparticles. As mentioned in the Introduction, lower removal rates are acceptable for the generation of ICs less than 10 nm.

**Figure 11:**
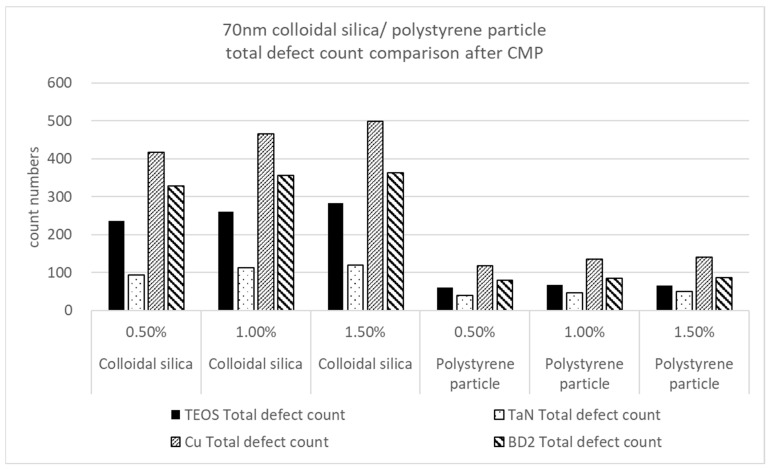
Total polishing defects for four kinds of material substrates in a comparison of colloidal silica and polystyrene according to nanoparticle solid contents of 0.5%, 1%, and 1.5% at a particle size of 70 nm.

A critical comparison of the total defect counts for the colloidal silica and polystyrene nanoparticles is demonstrated in Figure 11. We can see that the four kinds of materials on the wafer surface were significantly improved, reducing the total defect counts with polystyrene nanoparticles as a polishing abrasive. The total defect counts are also a function of the nanoparticle solid content. The greater the nanoparticle solid content is, the more defects are generated. We considered it reasonable to investigate the total defect number using mathematics, and we found that the inorganic silica’s rate of defect increase was faster.

**Figure 12:**
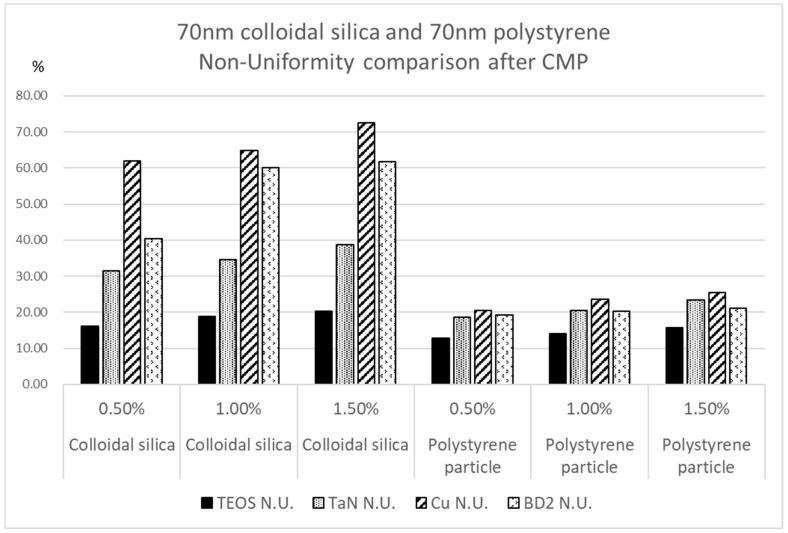
Non-uniformity (N.U.) value after CMP for four kinds of material substrates in a comparison of colloidal silica and polystyrene according to nanoparticle solid contents of 0.5%, 1%, and 1.5% at a particle size of 70 nm.

Another important criterion is uniformity. We call this evaluation value N.U. (non-uniformity), which is calculated as follows:

N.U.% = (MRR_max_ − MRR_min_)/2/MRR_average_ × 100% (1)

where MRR_max_ and MRR_min_ are the polishing material removal rates for the maximum value and the minimum value, respectively. The non-uniformity value is based on 49 checking points across the diameter of a wafer. For N.U., a lower value is better. We took a 1.5% nanoparticle solid content for a comparison with the polishing results. As Figure 12 clearly illustrates, the comparison shows that the polymer nanoparticles provided greater flatness to the wafer surfaces after polishing, and the values of N.U. are significantly lower than those of the inorganic nanoparticle silica. A good uniformity means that the next steps of IC fabrication, such as lithography or material deposition, can be conducted with greater ease. We can see that the non-uniformity values for polymer nanoparticle polishing are lower, and the difference between the four kinds of material substrates is also less significant. This minimal difference in the non-uniformity values meant that when we proceeded to polish the pattern wafers, the dishing or gaps between the materials substrates were smaller. In IC fabrication, material stacking and lithography are beneficial for better integration.

We showed that the polymer nanoparticles significantly improved the total defect counts and uniformity in the CMP polishing processes, although the polishing removal rates for the polymer nanoparticles were lower than those for traditional silica. Since there is a trend towards developing IC nodes that are smaller, with thinner material deposition on wafer surfaces, high polishing rates are optional, and polishing defects and uniformity become critical issues for the target and yield. Based on our results of the polishing defects, polymer nanoparticles exhibit elasticity between the wafer surface and the rotation polishing pad during CMP processes. The polymer nanoparticles move with the rotation pad and are compressed between the polishing pad and wafers. From the microscopic point of view, they are deformed due to their elasticity. This elasticity reduces the defects caused by direct collision impact because of the damping effect. Compared with traditional silica polishing particles, polymer nanoparticles have improved defect types and result in a lower total number of polishing defects. This is a tremendous improvement for the CMP industry.

### 3.2. Effect of Polishing Particle Shape

**Figure 13:**
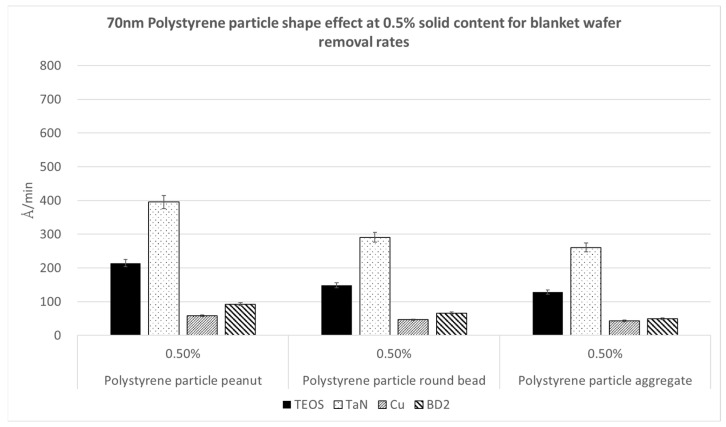
Polymer particle shape effect on removal rates at a 70 nm size and 0.5% nanoparticle solid content.

**Figure 14:**
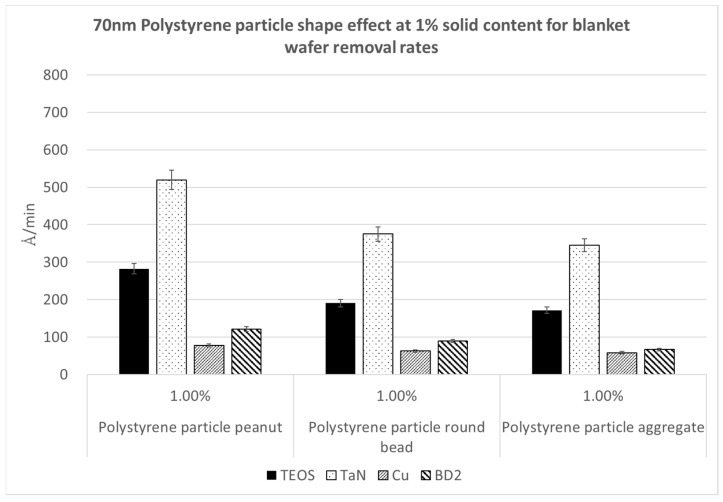
Polymer particle shape effect on removal rates at a 70 nm size and 1% nanoparticle solid content.

**Figure 15:**
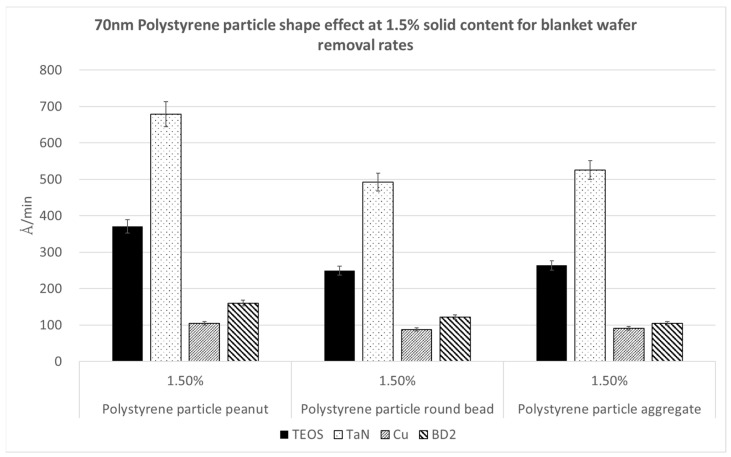
Polymer particle shape effect on removal rates at a 70 nm size and 1.5% nanoparticle solid content.

Figure 13, Figure 14 and Figure 15 demonstrate the removal rates for the 70 nm PS nanoparticles with different shapes, namely the peanut type, round bead, and aggregated long string, for four kinds of wafer substrates of TEOS, TaN, Cu, and BD2. We repeated these polishing tests using three nanoparticle solid contents of 0.5%, 1%, and 1.5%, in order to see whether the trends were the same. We can observe that in each figure, the same nanoparticle solid content shows the same trend in the polishing rates for the kinds of particle shapes. The peanut-shaped nanoparticles have the highest polishing removal rates.

**Figure 16:**
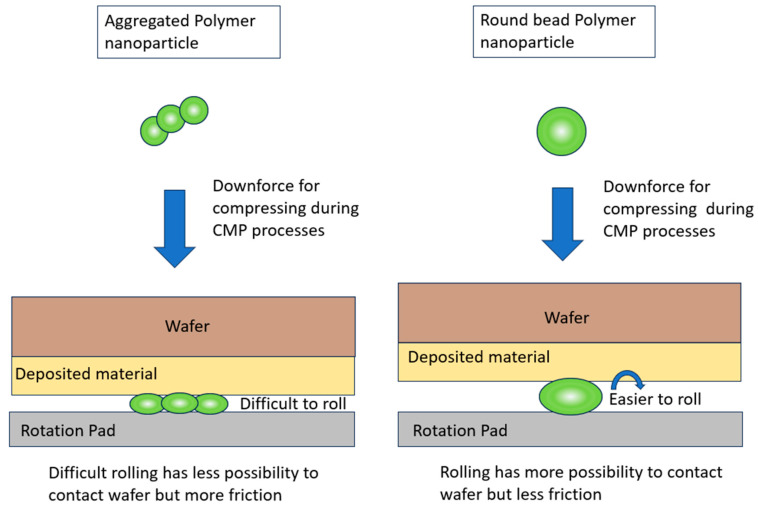
Motion behavior of round bead polymer nanoparticles and aggregated polymer nanoparticles during CMP processes.

According to the previous studies and theories of CMP removal rate mass transfer, CMP performance is related to each polishing particle’s contacting behavior with the wafers [28]. Polymer nanoparticles are evaluated based on their physical appearance, and their appearance is associated with their degree of freedom of motion and movement on wafer surfaces. A higher degree of freedom for motion means that the probability of mass transfer of the polished particles’ contacting position on the wafer substrates is higher. Nanoparticles of the long string aggregation have a lower degree of freedom for motion and higher friction due to a lack of rolling. As one would expect, the round-shaped particle can roll more easily on the wafer surface, with more opportunity to make contact with the wafer. For the aggregated particle, movement on the wafer surface is more difficult, offering less opportunity for wafer contact. However, the rolling particle has less friction than the aggregated particle, as illustrated in Figure 16. Therefore, the peanut-shaped nanoparticles balance these two factors and the individual forces of the respective nanoparticles, and showed the highest polishing removal rates. This optimized polishing efficiency is balanced by the medium degree of freedom of the peanut-shaped polymer nanoparticles.

**Figure 17:**
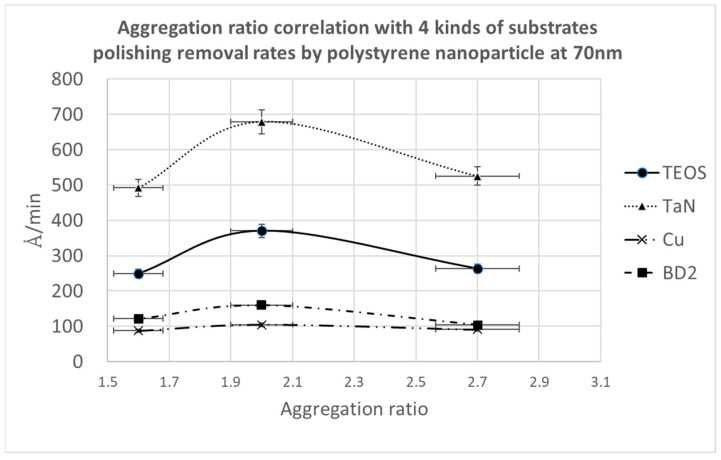
Aggregation ratio correlation with four kinds of substrates’ polishing removal rates, with a polystyrene nanoparticle size of 70 nm.

Since particle shape is correlated with the freedom of nanoparticle motion, we describe the particle shape with the aggregation ratio defined as D2/D1, in which D2 and D1 are the secondary and primary particle sizes. After the calculation, we can define particles with a value below 1.6 as having a round shape. A value of 2.0 indicates a peanut shape. A value above 2.7 indicates an aggregated long string shape. Figure 17 illustrates the polishing removal rate as a function of the aggregation ratio. It is clear that a value of 2.0 for the aggregation ratio (i.e., the peanut shape) can provide the maximum removal rates.

**Figure 18:**
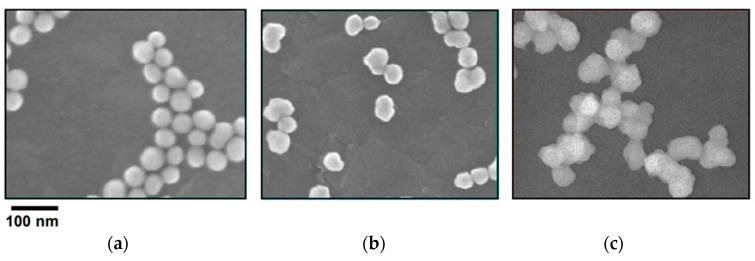
Nanoparticle shapes in SEM (JEOL JSM-IT800) pictures: (**a**) round bead shape of AP-70R; (**b**) peanut shape of AP-70P; (**c**) aggregated long string shape of AP-70A.

Through SEM verification, as shown in Figure 18, we see the appearance of the particle shapes: (a) the round bead shape, (b) peanut shape, and (c) aggregated long string shape.

### 3.3. Polymer Nanoparticle Size Effect

**Figure 19:**
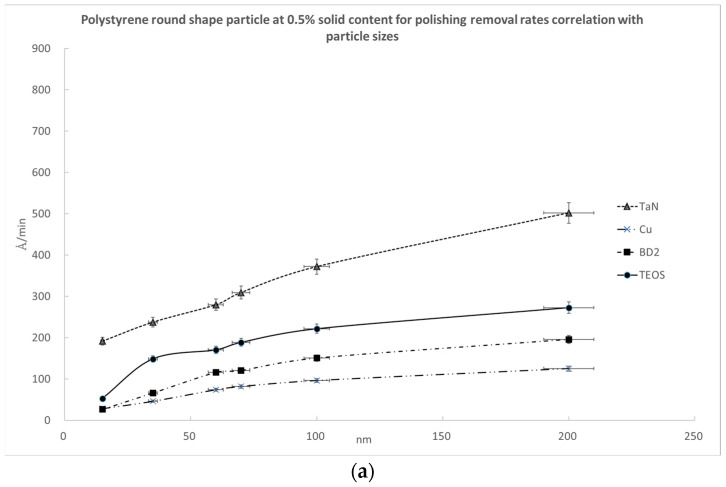

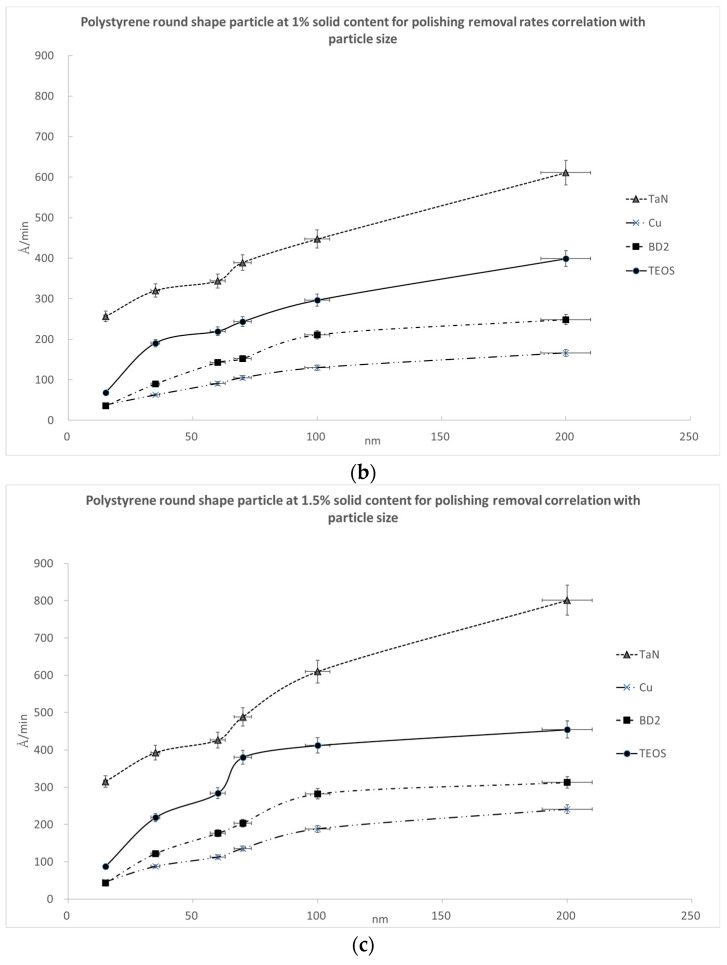
The polishing removal rates as a function of polystyrene nanoparticle size for four kinds of substrates at nanoparticle solid contents equal to (**a**) 0.5%, (**b**) 1%, and (**c**) 1.5%.

In Figure 19, we plotted the variation in the polishing removal rates with the different sizes of the round-shaped polystyrene nanoparticles for the four kinds of substrates at (a) 0.5%, (b) 1%, and (c) 1.5% polymer nanoparticle solid contents. As expected, the polishing results show that the larger polishing nanoparticles have higher polishing removal rates among the four kinds of wafer substrates.

**Figure 20:**
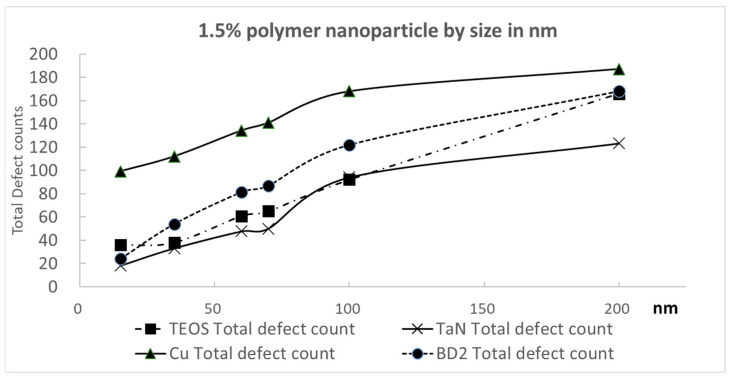
Total defect counts as a function of polystyrene particle size for round-shaped particles with a 1.5% nanoparticle solid content for four different kinds of substrates.

In Figure 20, we plotted the variation in total defect counts for different sizes of round-shaped PS nanoparticles at a 1.5% solid content for the four kinds of substrates. Since the polymer nanoparticle polishing defects were already quite low compared with those of the inorganic nanoparticles, we used the 1.5% polymer nanoparticle solid content as a representative value to magnify this difference in a comparison. We can see that the larger polymer nanoparticles showed more polishing defects for all four kinds of the wafer substrates. The polishing defect number can also be a function of the polymer nanoparticle size. The increasing slopes do not show steep trends.

**Figure 21:**
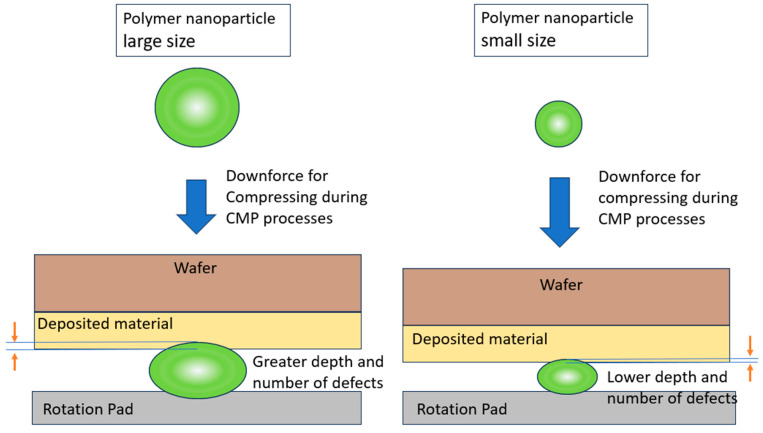
Larger polymer nanoparticle generating higher removal rates and more defects.

The polishing data show that the larger the size of the polishing particles is, the higher the mechanical force on the polishing removal rate will be. The larger nanoparticle size has a higher mechanical force because larger particles efficiently scoop more materials from the wafer surfaces, just as a larger excavator digs up more dirt from the earth than a smaller one. However, the larger polymer nanoparticles show a trend of increasing numbers of scratch defects produced during polishing. The inorganic nanoparticles have even more aggregated large particles to cause more severe defects. Therefore, the appropriate particle size is also important for the optimization of CMP abrasive slurries, in order to balance this trade-off factor. Higher polishing removal rates increase the manufacturing speed for increased throughput, because the polishing time is reduced; however, the increased number of scratch defects reduce the wafer yield in IC chip manufacturing. As illustrated in Figure 21, the differently sized nanoparticles show different polishing behaviors. A larger nanoparticle provides higher mechanical force, affecting its removal rates and total defect counts.

### 3.4. Effects of Various Copolymer Nanoparticle Molecules on CMP Polishing Performance

**Figure 22:**
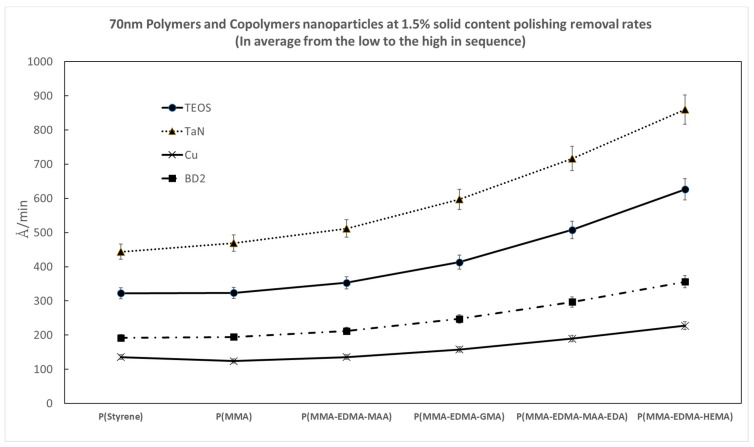
A comparison of the polishing removal rates for various polymer nanoparticle molecules on four kinds of wafer substrates at a particle solid content equal to 1.5% and particle size of 70 nm.

**Figure 23:**
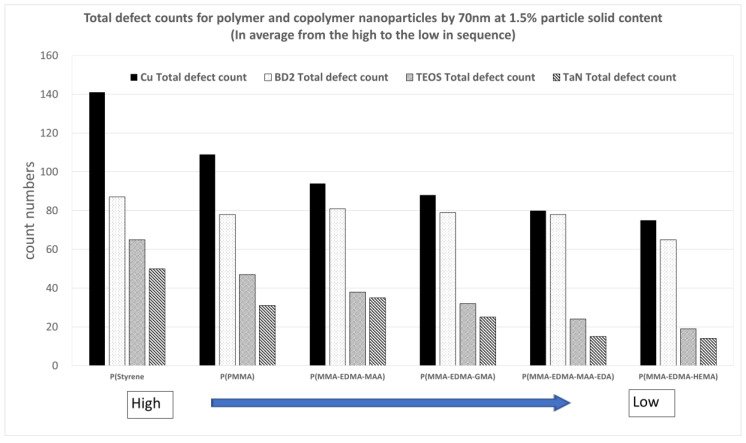
A comparison of the total defect counts for various polymer nanoparticle molecules on four kinds of wafer substrates at a particle solid content equal to 1.5% with a particle size of 70 nm.

In order to explore the effect of the molecular type of polymer nanoparticles on the resulting CMP polishing performance, Figure 22 and Figure 23 show comparisons of the polishing removal rates and defect counts using various nanoparticle molecules, including polystyrene, P(MMA), and copolymer molecules of P(MMA-EDMA-MAA), P(MMA-EDMA-GMA), P(MMA-EDMA-HEMA), and P(MMA-EDMA-MAA-EDA), for four kinds of wafer substrates at a particle solid content equal to 1.5%. It is interesting to note that the copolymer nanoparticles exhibited higher removal rates and fewer defects.

**Figure 24:**
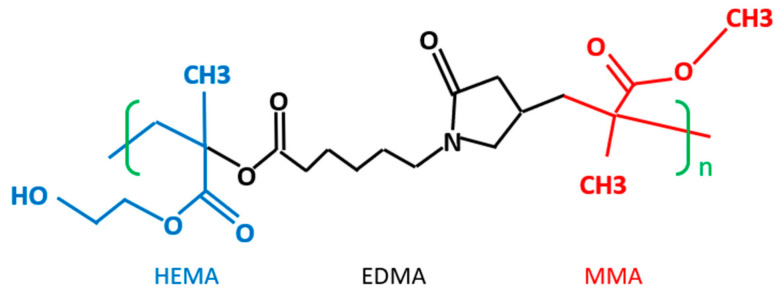
Triblock copolymer of P(HEMA-EDMA-MMA).

Based on the polishing performances, we believe that the different molecules of the copolymers exhibited different elasticity behaviors during the CMP processes. Rebound force is generated from polymer nanoparticles that are compressed and squeezed during CMP polishing. Their higher elasticity generates stronger rebound forces, and then this higher rebound force provides higher activity and motion frequency for polishing rate enhancement to remove material from the wafer surfaces. Meanwhile, the higher elasticity provided by the different copolymer molecules provides a greater damping effect to prevent direct collision impact on the wafer surfaces for a reduction in the number of total polishing defects. When we observe the molecule structure of P(MMA-EDMA-HEMA), we glean that this triblock copolymer may form a rigid area phase with the EDMA, from the medium-hard MMA phase to the soft area HEMA phase, as illustrated in Figure 24, subsequently forming some soft and hard combination micelles that generate unique morphological properties of the polymer nanoparticles [32]. This kind of copolymer nanoparticle, through a combination of physical properties, can generate a greater damping effect when the copolymer nanoparticles are compressed between the wafer and polishing pad during CMP processes.

### 3.5. JMP^®^ (SAS Institute) Statistic Software Analysis of the Polishing Data of Polymer Nanoparticles

**Figure 25:**
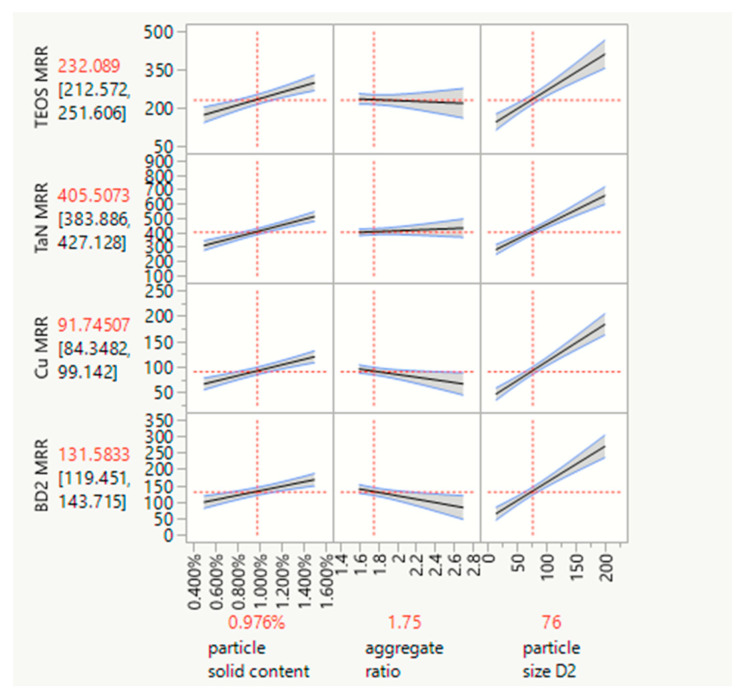
Removal rates for the four kinds of blank wafer substrates for TEOS, TaN, Cu, and BD2, showing correlations with the nanoparticle solid content, aggregation ratio, and particle size based on JMP^®^ software analysis.

**Figure 26:**
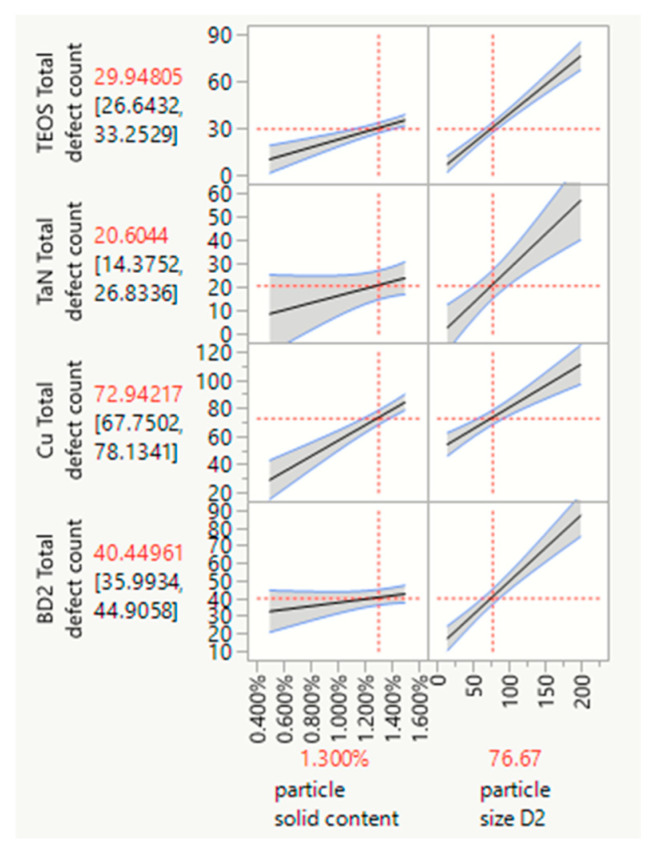
Defects counts for the four kinds of blank wafer substrates for TEOS, TaN, Cu, and BD2, showing correlations with nanoparticle solid content and particle size based on JMP^®^ software analysis.

The powerful commercial JMP^®^ (SAS institute) statistical analysis software can be used to efficiently analyze experimental data [33]. Regarding polishing removal rates and defect counts, it enables statistical analysis and shortens research and development times. Figure 25 displays the results of our analysis, showing how the removal rates for the four kinds of substrates are correlated with the polymer nanoparticle solid content, aggregation ratio, and particle size. Figure 26 displays the results showing how the total defect counts are correlated with the polymer nanoparticle solid content and particle size. We can observe the removal rate trend and the reliance interval of each factor. Meanwhile, moving the red center point to the removal targets, we can obtain the particle size, particle solid content, aggregation ratio target value, and range. Therefore, we can conclude that a 70~100 nm size, 1~1.5% nanoparticle solid content, and peanut shape are the best choices for an optimized polymer nanoparticle.

### 3.6. A Comparison of Pattern Wafer Polishing Results between Colloidal Silica and Polymer Nanoparticles

**Figure 27:**
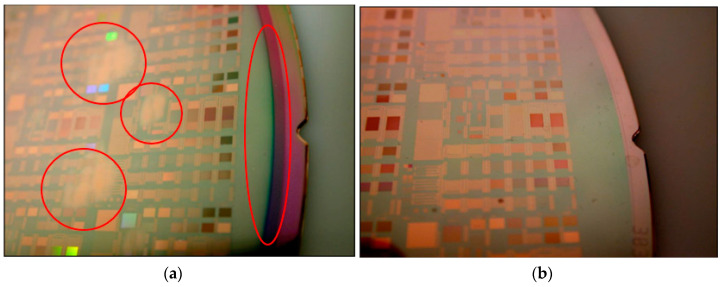
Polishing pattern wafers for (**a**) 70 nm peanut-shaped silica nanoparticle formulation slurry and (**b**) 70 nm peanut-shaped polymer nanoparticle formulation slurry.

Figure 27a,b show typical polishing patterns using traditional silica and polymer nanoparticle slurries based on an optimized chemical formulation. This comparison is based on 70 nm peanut-shaped polystyrene and 70 nm colloidal silica. Obvious differences can be seen on the wafer surfaces. The red circles mark the areas showing more uneven polished wafer surface materials. The conventional silica nanoparticles have a poor uniformity for planarization. In using optimal polymer nanoparticles formulated into a slurry for polishing, the uniformity and planarity were greatly improved.

## 4. Conclusions

For CMP processes, there are three important results regarding polishing removal rates, defect counts, and uniformity for the identification of a suitable polishing slurry for manufacturing integrated chip wafers. The major component in the polishing slurry is its abrasive particles, and we can see that polymer nanoparticles, as abrasives, can significantly improve the total defect counts for the yield and provide better uniformity for IC fabrication processes.

A recent development in IC wafer fabrication is the reduction in defects, especially when the IC line and space node are gradually reduced down to 5 nm, 3 nm, and even 1 nm, from the limitation of Moore’s law. The standards required for the type and number of defects are critical issues. The goal of reaching zero defects is practically the only direction through which wafer fabrication may generate future profits and competitiveness in the IC fabrication industry. Polymer nanoparticles produce fewer polishing defects than inorganic nanoparticles. Fewer polishing defects means higher yields and greater competition for IC manufacturers; meanwhile, polymer nanoparticles also show better uniformity than inorganic nanoparticles. In this study, we systematically demonstrated, for the first time, the effects of polymer nanoparticles’ behavior and physical properties on CMP performance.

We conclude that, indeed, polymer particles reduce the total polishing defect count, and round bead-shaped and peanut-shaped particles with a size of 70~100 nm at 1% polymer nanoparticle solid content provide sufficient polishing removal rates and good total defect counts within the required targets, as well as good uniformity. Another important finding is that copolymer nanoparticles show higher removal rates, and the total defect counts can also be reduced. The elasticity of copolymer nanoparticles leads to rebound behavior between the wafers and polishing pad, increasing their activity for the enhancement of polishing removal rates; meanwhile, more elastic polymer nanoparticles have greater damping effects for defect reduction. The defect-free performance CMP slurry by optimized polymer abrasives and chemical formulations is a key target for high yield manufacture competitive of the IC wafer fabrication industry.

## Figures and Tables

**Table 1:** A series of nanoparticles for CMP polishing tests.

Item	Type	Material	Size (D2)	Size (D1)	Shape
A1	Inorganic	Colloidal silica	70 ± 5 nm	41 nm	Round
AP-70R	Polymer	P(Styrene)	70 ± 5 nm	41 nm	Round
AP-70P	Polymer	P(Styrene)	70 ± 5 nm	36 nm	Peanut
AP-70A	Polymer	P(Styrene)	70 ± 5 nm	32 nm	Aggregated
AP-015R	Polymer	P(Styrene)	15 ± 5 nm	9 nm	Round
AP-035R	Polymer	P(Styrene)	35 ± 5 nm	21 nm	Round
AP-060R	Polymer	P(Styrene)	60 ± 5 nm	35 nm	Round
AP-100R	Polymer	P(Styrene)	100 ± 5 nm	59 nm	Round
AP-200R	Polymer	P(Styrene)	200 ± 5 nm	118 nm	Round
AP12-70R	Polymer	P(MMA)	70 ± 5 nm	41 nm	Round
AP14-70R	Copolymer	P(MMA-EDMA-MAA)	70 ± 5 nm	41 nm	Round
AP15-70R	Copolymer	P(MMA-EDMA-GMA)	70 ± 5 nm	41 nm	Round
AP18-70R	Copolymer	P(MMA-EDMA-MAA-EDA)	70 ± 5 nm	41 nm	Round
AP20-70R	Copolymer	P(MMA-EDMA-HEMA)	70 ± 5 nm	41 nm	Round

**Table 2:** Polishing recipe and equipment accessories.

Platen	Head	Downforce for Head	Pad Type	Polishing Time	Pad Clean Disk & Procedure	Slurry Flow Rate
87 rpm	83 rpm	2 psi	IC1010	1 min	3M A189L	200 mL/min
					Ex-situ 5 lbf.	

**Table 3:** Polishing wafer types and their original thicknesses.

Size	Wafer ID	Materials	Deposit Method	Thickness
8 inches	TEOS	Tetraethyl o-silicate	PECVD	5000 Å
8 inches	TaN	Tantalum Nitride	Sputtering and annealing	2000 Å
8 inches	Cu	Copper	Electroplating	5000 Å
8 inches	BD2	Porous dielectric	Spin coat and curing	5000 Å

## Data Availability

The data presented in this study are available on request from the corresponding author.
